# Barriers to effective health care for patients who have smell or taste disorders

**DOI:** 10.1111/coa.13818

**Published:** 2021-06-15

**Authors:** Stephen Ball, Duncan Boak, Joanne Dixon, Sean Carrie, Carl M. Philpott

**Affiliations:** ^1^ Newcastle University Faculty of Medical Sciences Newcastle upon Tyne UK; ^2^ Fifth Sense Barrow‐on‐Furness UK; ^3^ Newcastle upon Tyne Hospitals NHS Foundation Trust Newcastle upon Tyne UK; ^4^ The Norfolk Smell & Taste Clinic (Norfolk & Waveney ENT Service) James Paget University Hospital Gorleston UK; ^5^ Norwich Medical School University of East Anglia Norwich UK

**Keywords:** neurology, olfaction disorders, otorhinolaryngology, parosmia, primary health care, smell dysfunction

## Abstract

**Objectives:**

Smell/taste disturbances are a common but underrated, under‐researched and under treated sensory loss and an independent risk factor for reduced longevity. This study aimed to characterise the experience of patients with these disorders in seeking help.

**Design:**

The study was designed by patients together with clinicians through a dedicated workshop and conducted as a cross‐sectional survey to capture experiences in public and private healthcare settings internationally.

**Setting:**

Primary, secondary and tertiary care.

**Participants:**

Any members of the public self‐reporting a smell/taste disorder were invited to participate.

**Main outcome measures:**

The survey captured information including experience of getting consultations and referrals to medical professionals, treatments offered, costs incurred and related problems with mental health.

**Results:**

Of 673 participants; 510 female, 160 male, three not stated, self‐reported aetiology included sinonasal disease (24%), idiopathic (24%) and post‐viral olfactory dysfunction (22%); true gustatory disorders were typically rare. Failure of medical professionals to recognise the problem was a key concern ‐ 64%, 76% and 47% of GPs, ENT specialists and Neurologists acknowledged, respectively. Other issues included repeated ineffective treatments, difficulties getting referrals to secondary/tertiary care, mental health problems (60%) and a mean personal cost of £421 to seeking advice and treatment. Whilst the participants were self‐selecting, however, they do represent those who are seeking help and intervention for their disorders.

**Conclusion:**

There is an unmet need for these patients in accessing health care including a clear need to improve education of and engagement with the medical profession in Otorhinolaryngology, General Practice and other specialties, in order to remove the current barriers they face.

**Keypoints**

- Smell/taste disorder patients have difficulty getting their disorder recognised.
- Key problems are a lack of onward referral and repeated ineffective treatments.
- Neurologists are least likely to acknowledge their disorders.
- Mental health problems secondary to their disorder are very common.
- Patient engagement in research priorities is critical and being addressed through a Priority Setting Partnership.

## 1 INTRODUCTION

### 1.1 Background and rationale

Smell is the forgotten sense; even when facing a problem with their sense of smell, patients often struggle to get recognition, let alone diagnosis or treatment from healthcare professionals. Based on comparative data from the Royal National Institutes for the Blind and the Deaf, olfactory disorders are as common as profound hearing loss and blindness affecting an estimated 5%‐20% of the population.^1^ Common causes of olfactory disorders include chronic rhinosinusitis, post‐viral olfactory loss and post‐traumatic olfactory loss^2^ as well as it being present in the majority of cases of Parkinson's disease and common in Alzheimer's disease.^3^ We have also witnessed the rise of sudden onset anosmia as a marker of COVID‐19 infection caused by the SARS‐CoV‐2 virus.^4^, ^5^, ^6^, ^7^, ^8^, ^9^, ^10^, ^11^ It remains to be seen how many new cases of lasting olfactory loss will arise from those afflicted by the pandemic, but it is possible that over 6 million people globally now have symptoms that last beyond 4 weeks based on WHO infection rates.

Recent population studies have now identified anosmia as an independent risk factor for shortened longevity, even after controlling for dementia and cardiovascular disease.^12^, ^13^, ^14^, ^15^ It is not clear why this is so but may suggest that the olfactory system acts as a barometer of environmental impact on the central nervous system as a whole. As this phenomenon has been observed in several countries it clearly demonstrates that olfactory disorders deserve to receive greater attention than they currently do.

Taste is often thought to be lost by those affected by olfactory disorders due to the misperception of retronasal olfaction as a “taste” sensation. In reality only a small percentage of people reporting a problem with their sense of smell or taste experience a true gustatory disorder, but due to this common misperception alongside the need to be representative of all patients with chemosensory disorders, it is always important to encapsulate both senses within any work of this kind.

Fifth Sense, the UK charity for people affected by smell and taste disorders, was founded in 2012 when authors DB and CP met and agreed on the need for patient advocacy to tackle the unmet needs of patients affected by olfactory disorders. Since then, with the help of a growing membership, we have been able to characterise the significant impact of olfactory disorders on those affected^16^, ^17^ and we have also become aware of the frustrations many members in their attempts to seek medical help and getting their sensory loss taken seriously.^17^, ^18^, ^19^ In 2019, Fifth Sense was awarded a National Lottery Grant for £238 815 to enable it to develop and grow its work, including the establishment of a network of patient support hubs.

### 1.2 Objectives

Following on from the above, this study aimed to characterise the details of the difficulties faced by patients with olfactory and gustatory disorders in accessing health care as a patient and public co‐production.^20^

## 2 MATERIALS AND METHODS

### 2.1 Study design

**FIGURE 1:**
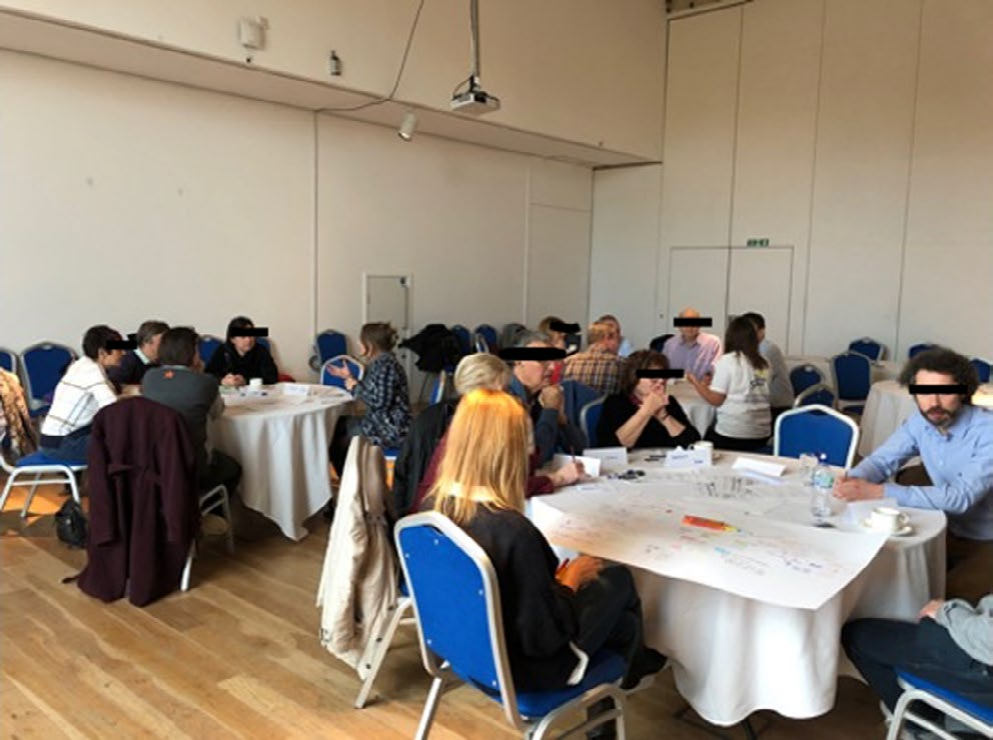
Workshop in progress—Group discussions between participants, Fifth sense members, clinicians, researchers and artists to expand on the themes of the day—“mapping the patient journey & its challenges”

**FIGURE 2:**
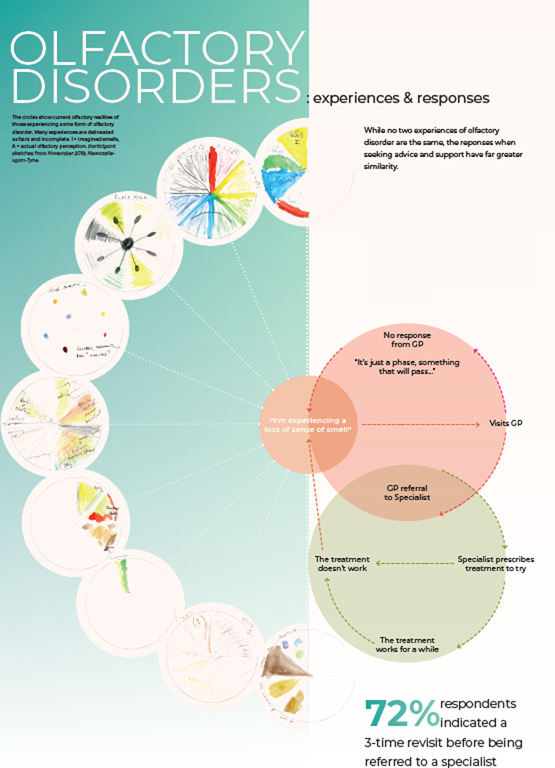
“Customer journey”—workshop activity

The study was designed as a cross‐sectional survey of the experience of people affected by olfactory disorders in accessing medical care. A survey questionnaire was developed using a focus group meeting of the public, patients and clinicians (Figures 1 and 2). The survey was then set live online and ran for 16 weeks. It was promoted via social media internationally. As the survey was anonymous and considered to be service evaluation, there was no ethical approval sought in line with the Health Regulation Authority guidance: http://www.hra‐decisiontools.org.uk/research/docs/DefiningResearchTable_Oct2017‐1.pdf.

### 2.2 Setting

The survey was open to anyone globally with access to the world wide web and declaring themselves an affected individual and was promoted through social media channels including the Fifth Sense website, and its Facebook and Twitter accounts. The survey was launched via the website with an introduction found in Appendix 1.

### 2.3 Participants

#### 2.3.1 Eligibility criteria

All members of the public self‐reporting a loss or disturbance of olfaction and/or gustation were entitled to participate in the survey.

#### 2.3.2 Sources and methods of selection of participants

Survey participants were able to access the survey themselves free of charge via the web‐based platform SurveyMonkey. Participants were self‐selecting and could participate from any country internationally.

### 2.4 Data sources/management and variables

The survey asked for basic demographics including age and sex. Participants were asked to declare the underlying cause for their smell loss. Further questions explored participants use of medical services, costs borne in doing so and any resistance encountered. See Appendix 1 for details.

### 2.5 Bias

We aimed to reduce bias in responses by making the survey widely available but inevitably, those who are not online or have access to the aforementioned social media would not have seen this opportunity. Although the survey was initiated in the UK and Fifth Sense membership is predominantly UK based, the international availability aimed to derive a broader healthcare view across other healthcare systems.

### 2.6 Study size and statistical methods

As this was an exploratory study, no sample size was set. Due to the nature of the study, descriptive statistics only have been utilised in reporting the survey data.

## 3 RESULTS

### 3.1 Participants

A total of 673 participants recorded information on the survey during the study period. Not all 673 participants responded to every question, so percentages below are expressed with the total number of responses as the denominator. For reference, there are currently 3000 people registered as members of Fifth Sense.

### 3.2 Descriptive data

**FIGURE 3:**
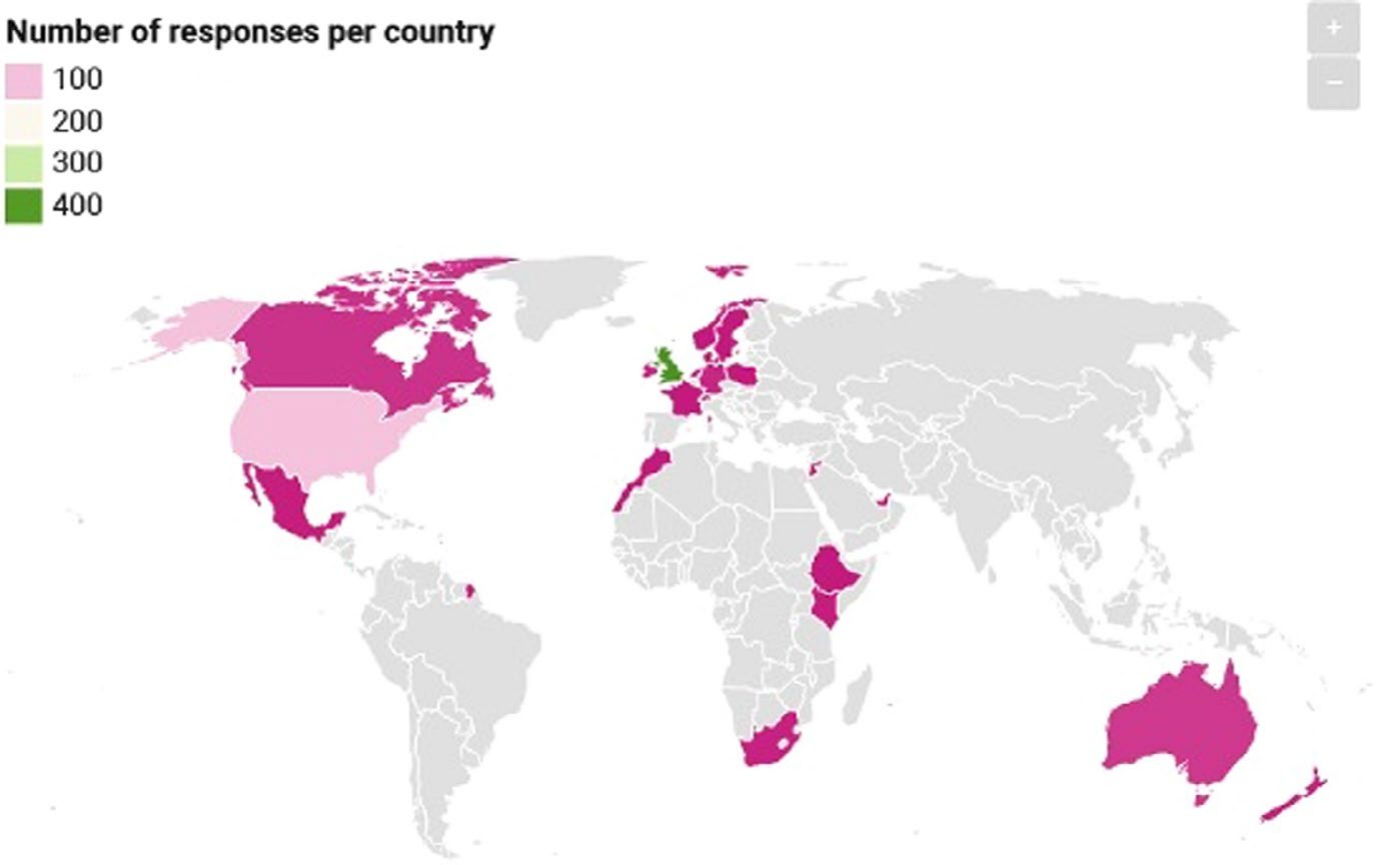
Geographic distribution of survey respondents

**FIGURE 4:**
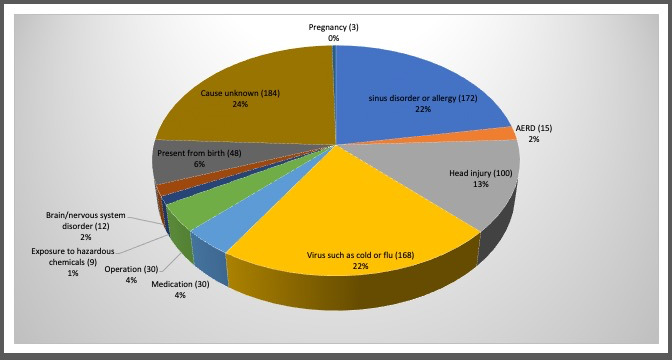
Aetiology of respondents

Of the 673 participants, 510 were female and 160 were male; three preferred not to state their gender. The age of participants ranged from 10 to 88, with a mean age of 56 and a mode age of 63. The geographic distribution of participants can be seen in Figure 3 with 469 (70%) reporting their location as being in the UK. The aetiology reported for participants can be seen in Figure 4 with 28% reporting chronic rhinosinusitis, allergic rhinitis or Aspirin/Non‐Steroidal Exacerbated Respiratory Disease (A/NERD) and 25% reporting post‐viral olfactory loss (PVOL). The range of duration of reported olfactory disorders was 1 month to 67 years with a mean of 13 years and a mode of 2 years.

### 3.3 Main results

#### 3.3.1 Recognition of the olfactory disorder as a significant problem

Getting recognition from doctors that their condition is a significant problem for them was challenging for some participants, with 64% reporting that their GP positively recognised their disorder and 76% reporting recognition by an otorhinolaryngologist; for those who had seen a neurologist, only 47% reported that they felt their disorder had been recognised and for those seeking a private specialist opinion, 66% (Table 1a, 1b, 1c).

**TABLE 1a:** Survey responses (part 1)

Question	Yes	No
	n (%)	n
Have you been seen by a		
GP?	507 (95)	27
ENT specialist?	444 (87)	68
Neurologist?	112 (34)	221
Private provider?	130 (40)	199
Have they recognised your smell/taste disorder is a problem to you?		
GP	319 (64)	178
ENT specialist	304 (75)	104
Neurologist	58 (47)	66
Private provider	99 (66)	50
Have you been prescribed any treatment?		
GP	195 (40)	293
ENT specialist	238 (54)	199
Neurologist	11 (9)	114
Private provider	69 (47)	78
Have they provided you with any useful information or advice about your condition?		
GP	35 (7)	456
ENT specialist	149 (33)	298
Neurologist	17 (13)	111
Private provider	41 (28)	107
Have you been prescribed the same treatment by them on more than one occasion?		
GP	123 (31)	269
ENT specialist	131 (37)	223
Neurologist	2 (2)	85
Private provider	34 (28)	86
Has any treatment prescribed by them improved your sense of smell/taste?		
GP	30 (8)	349
ENT specialist	81 (22)	270
Neurologist	2 (2)	90
Private provider	25 (20)	99

**TABLE 1b:** Survey responses (part 2)

Question	Yes n (%)	No
Have you been referred to an ENT Specialist by your GP/Neurologist?	389 (71)	158
Were you given a choice of location?	148 (34)	284
Were you given a choice of Specialist?	87 (20)	348
Did you use information obtained from Fifth Sense?	134 (29)	330
Did you request a referral?	265 (54)	227
Were you offered a referral?	251 (53)	225
Was your case recognised as needing a referral?	274 (60)	185
Was your case declined by the local Clinical Commissioning Group (CCG) so your GP was unable to help you?	12 (4)	294
Has information provided by Fifth Sense helped you in your efforts to get medical advice?	223 (40)	339
Do you consider that your smell/taste disorder has affected your quality of life?	548 (98)	14
Have you suffered from anxiety or depression as a result of your smell disorder?	340 (61)	221
If yes to anxiety or depression, have you?		
Taken any medication prescribed by your GP? (eg antidepressants, sedatives, anxiolytics)	102 (30)	238
Taken an over‐the‐counter medicine?	42 (12)	298
Taken an alternative medicine remedy?	75 (22)	265
Received counselling?	73 (21)	267
Do you think your ability to smell/taste has improved since you first encountered problems with it?	138 (27)	376
Do you feel that this is as a result of medical advice and/or treatment?	89 (20)	353
Do you feel that this is the result of a complementary therapy such as smell training?	59 (14)	354

**TABLE 1c:** Survey responses (part 3)

	Mean	Range
How many appointments have you had in total with?		
GPs	5.6	0‐150
ENT specialist	4.6	0‐75
Neurologist	0.9	0‐54
Private provider	1.6	0‐50

#### 3.3.2 Prescription of treatment

In primary care, 195 respondents (40%) reported receiving a prescription related to their olfactory disorder. In secondary care, respondents reported receiving a prescription from 54% of otorhinolaryngologists and 10% of neurologists, respectively. For those who sought private consultations, 46% reported receiving a prescription. Repeated treatments were reported from 31% of GPs to 37% of otorhinolaryngologists. In terms of effectiveness of the treatment, 8% of GP prescriptions, 23% of ENT prescriptions, 2% of neurology prescriptions and 20% of private prescriptions were deemed by the patients to have helped improve their sensory deficit.

#### 3.3.3 Useful information and advice

In primary care, only 7% felt that they received useful information and advice, with a rise to 33% for ENT and 13% in neurology in secondary care and in the private sector only 28%.

#### 3.3.4 Healthcare consultations

The range of reported GP consultations for respondents was 0–150 with a mean of 5.8 and a mode of 1. In Otorhinolaryngology, the range was 0–75 with a mean of 4.6 and a mode of 1. For Neurology, this was much lower with a range of 0–54 and a mean of 0.9, and similarly, in private health care, the range was 0–50 with a mean of 1.7. Seventy‐one per cent of respondents reported being able to get a referral to Otorhinolaryngology and of these 34% were given a choice of location but only 20% a choice of specialist. Information from the Fifth Sense website informed 29% to guide their choice of referral centre with 54% having to request the referral themselves and 59% stating they felt their case was recognised as needing a referral. Only 4% of respondents reported having their case declined by their local Clinical Commissioning Group but 40% felt that Fifth Sense information had helped the process of getting medical advice.

#### 3.3.5 Travel and cost of healthcare appointments

Respondents were asked to estimate how far they had travelled in miles to seek information or treatment for their disorder and reported a range of 0 to 15 250 miles with a mean of 200 miles and a median of 30 miles. The personal cost of doing so showed a range of £0‐41 100 (highest figure in USA) with a mean of £421 and a median of £50. When analysed further by country, the USA has the highest mean cost per respondent at £2277, followed by Australia, Canada, United Kingdom and New Zealand (Table 2).

**TABLE 2:** Mean healthcare cost per country, where five or more responses received

	Average cost (GBP)	Number of responses
The United States	2277	121
Australia	1491	23
Canada	407	13
UK	375	477
New Zealand	190	5

#### 3.3.6 Improvement and treatment impact

Only 138 respondents reported an improvement of their disorder with 89 (17%) saying they felt as if this was the result of medical advice and/or treatment and 59 (11%) who felt that it was in response to smell training.

#### 3.3.7 Quality of life including mental health

All but 14 respondents reported an impact on their quality of life with 60% reporting either anxiety and or depression as a consequence of their sensory loss. Specific treatments reported included 102 taking GP‐prescribed antidepressants/sedatives/anxiolytics (15%), 42 taking over the counter remedies (6%), 73 receiving counselling (11%) and other alternative therapies used included acupuncture, marijuana use and meditation (4%).

#### 3.3.8 Overall patient perspective

An open comments section was included to supplement the quantitative data that provided some important insights from our participants' perspectives. The quotations were chosen by our patient co‐authors as representative of the typical experiences.

1. It is not really taken seriously. The attitude is almost “Well, at least you are not deaf or blind.” The effect on my daily life is not recognised.
2. I am low in mood. I hate eating and don't feel hungry. It affects my job and makes me feel unsafe from fire and gas leakages.
3. I feel that even ENT specialists do not see this problem which truly affects your quality of life as even a problem.
4. Feel like it's minimized by people and professionals who think it must be nice not to smell kids' dirty nappies or that you're only missing out on smelling flowers and cookies in the oven. It is a real issue ‐ gas hob, smoke, taste diminishes, lack of shared experience with family, and memories compromised.
5. Following treatment, I am able to smell again much of the time. It is so wonderful to be able to smell the ocean, to smell coffee brewing, to smell bacon or onions cooking. I can smell my husband's skin, or the soap he last used. I can smell the soap I use in the shower, which never ceases to amaze me. I can smell whether fruit is ripe or not. This is all incredibly wonderful, it adds such a richness to your life. Not to mention: I can smell gas, if the burner on the stove didn't turn on correctly, or paint, if a hallway is newly painted. I can smell if milk has gone sour. If I can smell these things I can protect myself from them better.

## 4 DISCUSSION

### 4.1 Key results

Our study serves to illustrate a number of significant issues faced by patients with chemosensory disorders. Firstly, a lack of recognition in the wider medical profession but still with 1 in 4 ENT specialists failing to convince their patients that they appreciated the impact of their olfactory disorder; this was even more noticeable in over half of neurologists encountered. In fact, it is evident that the experience of this patient group with neurologists was largely disappointing. Secondly, knowledge of appropriate treatments is lacking thus leading to no treatment or to repeated ineffective treatments being applied. This was further exacerbated by participants expressing little satisfaction with the usefulness of the advice given. Thirdly, accessing a specialist consultation was a challenge with 1 in 4 reporting difficulty in getting a referral. Due to the paucity of specialists dedicated to chemosensory disorders, respondents reported significant journeys and costs associated with that. Finally, mental health aspects of being affected by chemosensory disorders have been clearly highlighted and 15% reported taking prescribed medication for this, therefore, also emphasising an additional healthcare burden.

### 4.2 Limitations

The survey will not have been seen by those who are not online or do not have access to the aforementioned social media. This is likely to have disproportionately affect the older generations. Due to the origin of the survey in the UK and with Fifth Sense being a UK‐based charity, over two thirds of the respondents reflect their experience with the National Health Service setting in the UK. It is also possible that an unknown number of patients may have had a good response to treatment, but these cases will not be apparent if they are not Fifth Sense members or have chosen not to respond to the survey because they were happy with the outcome of their treatment. It may also be that the treatments applied were reasonable, but nonetheless proved ineffective in resolving or improving the olfactory disorder. The charity membership and survey respondents will also tend to be more likely to be those who have persistent symptoms and thus are more difficult to treat. That said, respondents are reflective of those in need and seeking care and attention and have been shown in our previous work to use NHS resources in other ways if their sensory loss(es) are not addressed.^16^ The survey also captured a retrospective perspective on those who had experienced difficulties with accessing healthcare.

The travel and cost issues demonstrated a significant spread of data; this may however reflect the small number of specialist centres seeing these patients and thus the distances and costs they face having to travel to them; the authors know of patients willing to travel to another continent for help and advice, even in countries with established health services free at the point of access.

Although the survey is based on self‐reporting and no psychophysical testing has been performed, this obviously would not reflect the nature of the core problem facing these patients; they are unable to access a clinician and smell/taste testing in the first place. For any children participants, the responses will reflect the views of the parents/guardians rather than the child.

### 4.3 Interpretation

The demographics and aetiology of study participants were in keeping with the typical female predominance seen in other studies and with sinonasal disease and post‐viral olfactory loss as leading causes.^21^, ^22^, ^23^, ^24^ The study also underlines the mental health impact of previous studies in those with olfactory disorders.^16^, ^17^, ^21^ However, this study makes a clear reflection on the paucity of services provided to this patient group and shows that the relatively poor engagement by the medical profession has changed little in nearly two decades.^25^ Our data would suggest this is clearly an issue in the UK healthcare setting, but international responses also suggest this is potentially a global issue with little emphasis placed on either the importance of these senses in everyday life or the consequences of losing them.

### 4.4 Generalisability

There is an unmet need for patients with olfactory disorders in accessing healthcare including engagement from the medical profession and signposting to appropriate information and treatment options.^26^ It remains to be seen as to whether the current wave of COVID‐19‐related smell loss as a result of the global pandemic will give rise to an increase in patients presenting with post‐viral olfactory loss but with an estimated rate of anosmia globally of 5% and hyposmia up to 20%, these disorders are common and engagement from the medical profession is not matching this. This underlines the raison d'être of Fifth Sense which amongst its strategic aims is the need to improve education of the role of these senses in everyday life as well as providing support for those affected by these disorders. To move this forward, Fifth Sense plans to work with the medical profession to not only provide patient support, but also to work with the wider body of stakeholders that need to be engaged in improving the current situation faced by these patients in accessing suitable care, including appropriate psychological support. As part of this initiative, Fifth Sense is leading the James Lind Alliance Priority Setting Partnership to determine what should be the top ten research priorities for those with smell and taste disorders https://www.jla.nihr.ac.uk/priority‐setting‐partnerships/smell‐and‐taste‐disorders/.

## REPORTING GUIDELINES

This study has been reported in line with the Strobe guidelines (see Appendix 2).

## Data Availability

Data can be made available on request to s.l.ball@ncl.ac.uk.

## APPENDIX 1 Introductory statement provided to participants

Fifth Sense is launching a survey to capture patients' experiences of navigating the healthcare system. We know that so many of you face real challenges in getting support, advice or treatment from your doctors, although there are success stories too. The survey has been designed to capture data that will highlight both these challenges and successes as part of our ongoing efforts to improve awareness amongst the medical profession and improve patient experience.

We believe that this is the first piece of research to focus specifically on this issue and we're very proud to be part of a multidisciplinary project team. Supported by a grant from Newcastle Medical School, Fifth Sense have partnered with Mr Sean Carrie and Stephen Ball from Newcastle Freeman Hospital and Newcastle University and Olfactory Mapmaker Kate McLean and her colleague Rachel Hancock. An event in Newcastle in November 2018 brought together Fifth Sense and Voice North members to share experiences and help with the design of the survey. Kate and Rachel made drawings that captured the issues discussed and asked guests to complete “smell wheels” to visually represent their own smell experiences.

The results of the survey will be used to:

1. Inform Fifth Sense's ongoing efforts to raise awareness of smell and taste disorders amongst the medical profession and the need for widespread education/training for healthcare professionals
2. Help us develop information aimed at both patients and healthcare professionals to help ensure that patients have the best possible experience when seeking medical advice
3. Help future efforts to improve the patient journey through the healthcare system for people with a smell/taste disorder
4. Provide data to support applications future research studies and projects
5. Kate and Rachel are designing a poster to accompany the results which will visually represent some of the challenges faced by patients
6. The results will be published in an appropriate medical journal and on the Fifth Sense website.

The survey is completely anonymous and should take no longer than 10‐15 minutes to complete. It is based on the UK healthcare system, but it should still be relevant if you are based in another country. We would like contributions from people outside the UK as this data may help to show that this is a global issue. We'd also like to hear from parents of a child with a smell/taste disorder who have sought medical advice as it's important that your voices are heard too.

## APPENDIX 2 STROBE Statement—checklist of items that should be included in reports of observational studies

**Table:**

	Item No	Recommendation	Page
Title and abstract	1	(*a*) Indicate the study's design with a commonly used term in the title or the abstract	1
		(*b*) Provide in the abstract an informative and balanced summary of what was done and what was found	1
Introduction			
Background/rationale	2	Explain the scientific background and rationale for the investigation being reported	2
Objectives	3	State specific objectives, including any prespecified hypotheses	3
Methods			
Study design	4	Present key elements of study design early in the paper	3
Setting	5	Describe the setting, locations, and relevant dates, including periods of recruitment, exposure, follow‐up, and data collection	4
Participants	6	(*a*) *Cohort study*—Give the eligibility criteria, and the sources and methods of selection of participants. Describe methods of follow‐up *Case‐control study*—Give the eligibility criteria, and the sources and methods of case ascertainment and control selection. Give the rationale for the choice of cases and controls *Cross‐sectional study*—Give the eligibility criteria, and the sources and methods of selection of participants	4
		(*b*) *Cohort study*—For matched studies, give matching criteria and number of exposed and unexposed *Case‐control study*—For matched studies, give matching criteria and the number of controls per case	
Variables	7	Clearly define all outcomes, exposures, predictors, potential confounders, and effect modifiers. Give diagnostic criteria, if applicable	4
Data sources/measurement	8*	For each variable of interest, give sources of data and details of methods of assessment (measurement). Describe comparability of assessment methods if there is more than one group	4
Bias	9	Describe any efforts to address potential sources of bias	4
Study size	10	Explain how the study size was arrived at	5
Quantitative variables	11	Explain how quantitative variables were handled in the analyses. If applicable, describe which groupings were chosen and why	N/A
Statistical methods	12	(*a*) Describe all statistical methods, including those used to control for confounding	5
		(*b*) Describe any methods used to examine subgroups and interactions	
		(*c*) Explain how missing data were addressed	
		(*d*) *Cohort study*—If applicable, explain how loss to follow‐up was addressed *Case‐control study*—If applicable, explain how matching of cases and controls was addressed *Cross‐sectional study*—If applicable, describe analytical methods taking account of sampling strategy	
		(*e*) Describe any sensitivity analyses	
Results			
Participants	13*	(a) Report numbers of individuals at each stage of study—eg numbers potentially eligible, examined for eligibility, confirmed eligible, included in the study, completing follow‐up, and analysed	5
		(b) Give reasons for non‐participation at each stage	
		(c) Consider use of a flow diagram	
Descriptive data	14*	(a) Give characteristics of study participants (eg demographic, clinical, social) and information on exposures and potential confounders	5
		(b) Indicate number of participants with missing data for each variable of interest	
		(c) *Cohort study*—Summarise follow‐up time (eg, average and total amount)	
Outcome data	15*	*Cohort study*—Report numbers of outcome events or summary measures over time	N/A
		*Case‐control study—*Report numbers in each exposure category, or summary measures of exposure	
		*Cross‐sectional study—*Report numbers of outcome events or summary measures	
Main results	16	(*a*) Give unadjusted estimates and, if applicable, confounder‐adjusted estimates and their precision (eg, 95% confidence interval). Make clear which confounders were adjusted for and why they were included	5‐7
		(*b*) Report category boundaries when continuous variables were categorized	
		(*c*) If relevant, consider translating estimates of relative risk into absolute risk for a meaningful time period	
Other analyses	17	Report other analyses done—eg analyses of subgroups and interactions, and sensitivity analyses	N/A
Discussion			
Key results	18	Summarise key results with reference to study objectives	8
Limitations	19	Discuss limitations of the study, taking into account sources of potential bias or imprecision. Discuss both direction and magnitude of any potential bias	9
Interpretation	20	Give a cautious overall interpretation of results considering objectives, limitations, multiplicity of analyses, results from similar studies, and other relevant evidence	10
Generalisability	21	Discuss the generalisability (external validity) of the study results	10
Other information			
Funding	22	Give the source of funding and the role of the funders for the present study and, if applicable, for the original study on which the present article is based	11
